# Reprogramming host histone modifications by bacterial pathogens

**DOI:** 10.1016/j.mocell.2026.100321

**Published:** 2026-01-23

**Authors:** Shira Zelikman, Sun-Ju Yi, Kyunghwan Kim

**Affiliations:** Department of Biological Sciences and Biotechnology, Chungbuk National University, Cheongju, Chungbuk, Republic of Korea

**Keywords:** Bacterial pathogen, Host and microbe interaction, Host histone modification

## Abstract

Bacterial pathogens have evolved sophisticated strategies to manipulate host cellular processes, ensuring survival, replication, and long-term persistence. Beyond classical immune signaling, emerging evidence highlights the central role of epigenetic regulation in host-pathogen interactions. Pathogens exploit host chromatin through 2 principal mechanisms: (1) direct modification of histones by bacterial effector proteins with intrinsic enzymatic activities and (2) indirect modulation of host epigenetic states through alterations in signaling pathways or cellular metabolism. These interventions alter post-translational histone modifications—acetylation, methylation, phosphorylation, and lactylation—thereby reshaping transcriptional programs to suppress antimicrobial responses, promote immune tolerance, or establish persistent infection. This review summarizes recent advances in understanding the dynamic interplay between bacterial virulence and host chromatin regulation, highlighting epigenetic reprogramming as a key determinant of infection outcomes.

## Graphical abstract

**Figure:**
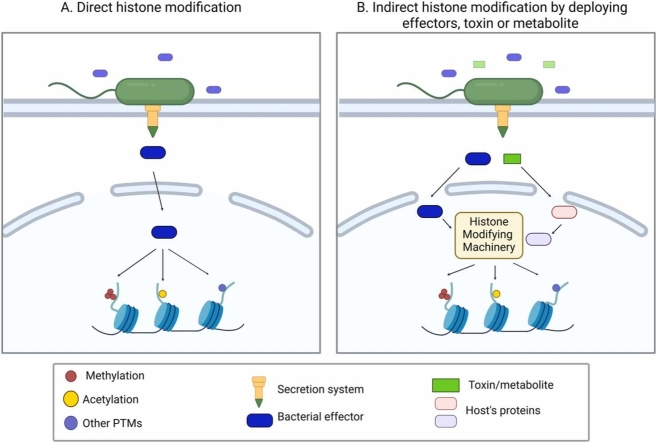

## INTRODUCTION

The dynamic interplay between mammalian host cells and microorganisms is central to understanding infectious disease pathology and immune defense mechanisms. Infections and host-pathogen interactions represent a fascinating exchange of molecular signals and cellular reprogramming. Deciphering these intricate relationships is essential for informing rational therapeutic design.

Microorganisms including bacteria, viruses, and fungi have evolved diverse strategies to manipulate host cellular processes, ensuring their survival, replication, and dissemination. In particular, bacteria deploy virulence-associated proteins, toxins, or metabolites to establish infection and circumvent host immunity by reprogramming host signaling pathways, metabolism, and epigenetic states (Brown et al., 2019, Woodhams et al., 2020). Although many of these alterations are transient, accumulating evidence suggests that pathogens can also induce long-lasting, heritable changes that play a critical role in the pathogenesis of infectious diseases and in the sustained persistence of pathogens within their hosts (Silmon de Monerri and Kim, 2014).

Accumulating evidence indicates that bacterial pathogens actively influence the host epigenome during infection. Rather than causing a global shutdown of transcription, bacteria selectively remodel host epigenetic landscapes to attenuate antimicrobial and inflammatory gene expression while maintaining host cell viability (Hamon and Cossart, 2008). Among epigenetic mechanisms, histone modifications are particularly responsive to infection, as they function at the interface of signaling pathways, metabolic state, and nuclear regulatory processes to control transcriptional output.

The impact of bacterial factors on host chromatin depends on how these factors are delivered to host cells. Bacterial secretion systems therefore represent a critical upstream determinant, defining whether virulence factors gain access to the host cytosol and nucleus or instead act extracellularly by engaging cell surface receptors and downstream signaling pathways. In this way, secretion routes constrain the modes through which bacterial factors can engage host chromatin-regulatory machinery.

**Fig. 1:**
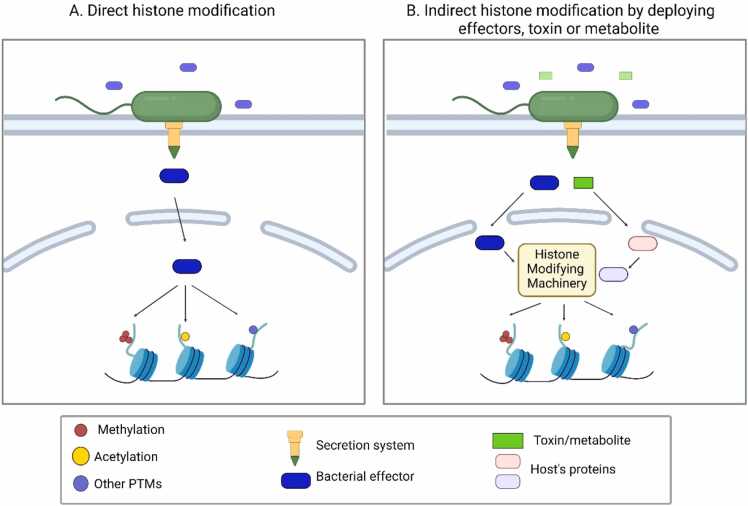
Mechanisms by which pathogenic bacteria modulate the host epigenetic landscape. Pathogenic bacteria employ 2 principal strategies to manipulate host histone modification and gene expression. (A) Direct histone modification, in which bacterial effectors with intrinsic enzymatic activity (eg, histone acetyltransferase or methyltransferase activity) directly modify host histones. (B) Indirect histone modification, in which bacterial effectors, toxins, or metabolites alter host signaling pathways or metabolic states, thereby affecting the activity, recruitment, or localization of host chromatin-modifying enzymes. These mechanisms result in changes in histone post-translational modifications and associated transcriptional responses during infection.

Within this context, bacterial modulation of histone modifications can be broadly categorized into 2 mechanistic strategies (Fig. 1). In direct epigenetic modulation, cytosol-delivered effectors exhibit intrinsic histone-modifying enzymatic activities and act directly on host chromatin. In indirect epigenetic modulation, bacterial effectors, toxins, or metabolites alter histone modification patterns by regulating the activity, localization, or expression of host chromatin-modifying enzymes. Both strategies converge on promoters and enhancers to alter chromatin accessibility and transcription factor-binding changes in host gene expression that favor bacterial survival and persistence (Bierne et al., 2012, Grabiec and Potempa, 2018).

Accordingly, this review first describes bacterial secretion systems as delivery routes that determine the cellular and subcellular distribution of virulence factors. It then examines how these delivery modes intersect with host histone modification pathways to reprogram transcriptional responses during infection, providing a framework for understanding how diverse bacterial strategies converge on the host epigenome.

## SECRETION SYSTEMS AS DELIVERY ROUTES FOR BACTERIAL FACTORS

**Fig. 2:**
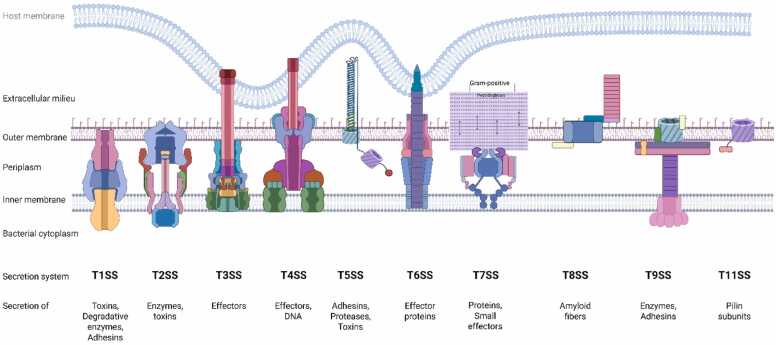
Schematic representation of bacterial secretion systems. Bacterial secretion systems are generally divided into 1-step and 2-step mechanisms. In 1-step systems (eg, T1SS, T3SS, T4SS, T6SS, and T7SS), proteins are transported directly from the cytoplasm to the outside or into host cells through large membrane-spanning complexes that cross both the inner and outer membranes. These systems enable rapid and contact-dependent delivery of effector proteins during infection or interbacterial competition. In contrast, 2-step systems (eg, T2SS, T5SS, T8SS, and T9SS) first use the Sec or Tat pathway to move proteins across the inner membrane into the periplasm, where they fold before being secreted through a separate outer membrane channel. These systems mainly function in secreting enzymes, toxins, and adhesins into the extracellular environment. T1SS, Type I secretion system; T3SS, Type III secretion system; T4SS, Type IV secretion system; T5SS, Type V secretion system; T6SS, Type VI secretion system; T7SS, Type VII secretion system; T8SS, Type VIII secretion system; T9SS, Type IX secretion system.

Bacterial pathogens employ diverse secretion systems (Fig. 2) to deploy virulence factors that manipulate host cellular processes. Secretion systems can be classified by whether bacterial factors are translocated into the host cytosol (cytosolic delivery systems) or instead released to act extracellularly, triggering host responses through surface receptors, uptake, or downstream signaling (Arechaga and Cascales, 2022, Baron, 2010, Beeckman and Vanrompay, 2010, Costa et al., 2015, Filloux, 2022, Maphosa et al., 2023, Tseng et al., 2009).

### Secretion Systems Enabling Cytosolic Delivery of Effectors

Several secretion systems enable bacteria to inject virulence factors directly into the host cytosol, providing immediate access to intracellular signaling pathways and, in some cases, the host nucleus. In Gram-negative, the Type III secretion system (T3SS), Type IV secretion system (T4SS), and Type VI secretion system (T6SS) are the best-characterized examples of this delivery mode.

T3SSs function as needle-like injectosomes that translocate effector proteins into host cells, where they rapidly modulate signaling cascades, cytoskeletal organization, and vesicular trafficking (Coburn et al., 2007, Cornelis, 2006, Cornelis, 2010, De Souza Santos and Orth, 2019, Galan et al., 2014, Hapfelmeier et al., 2005, Jennings et al., 2017). Similarly, T4SSs deliver protein or nucleoprotein complexes directly into host cells (Alvarez-Martinez and Christie, 2009, Christie et al., 2014, Costa et al., 2021, Zhang et al., 2012a) In *Helicobacter pylori,* T4SS-mediated injection of CagA rewires host signaling networks linked to inflammation and disease progression (Backert and Selbach, 2008, Backert et al., 2015, Cover et al., 2020, Terradot and Waksman, 2011). In *Legionella pneumophila*, the Dot/Icm T4SS translocates a large repertoire of effectors, including proteins that localize to the host nucleus or associate with chromatin, illustrating how direct cytosolic delivery facilitates extensive reprogramming of host cellular functions (Cambronne and Roy, 2007; Durie et al., 2020; Lockwood et al., 2022; Ma et al., 2024; Swart and Hilbi, 2020). T6SS primarily mediates interbacterial competition but, in certain contexts, can also deliver effectors into eukaryotic cells, thereby influencing host immune signaling (Cherrak et al., 2019, Ho et al., 2014, Russell et al., 2014, Sana et al., 2012, Silverman et al., 2012, Zhao et al., 2023).

In Gram-positive bacteria, the Type VII secretion system (T7SS) fulfills a comparable role. In *Mycobacterium tuberculosis*, T7SS-secreted effectors are essential for intracellular survival and include proteins that directly engage host nuclear processes (Malik et al., 2024, Simeone et al., 2009).

### Secretion Systems Releasing Extracellularly Acting Factors

In contrast, several secretion systems export bacterial factors without granting intracellular access, acting instead through extracellular mechanisms. These include the Type I, II, V, IX, and XI secretion systems, which predominantly secrete toxins, enzymes, or surface-associated proteins into the extracellular or pericellular environment (Cianciotto, 2005, Cianciotto and White, 2017, da Mata Madeira et al., 2016, Filloux, 2022, Gorasia et al., 2020, Grossman et al., 2021, Masi and Wandersman, 2010, Meuskens et al., 2019). The Type I secretion system (T1SS) is the simplest, exporting proteins directly from the cytoplasm to the extracellular environment in a single step (Masi and Wandersman, 2010, Morgan et al., 2017, Spitz et al., 2019, Thomas et al., 2014). In contrast, the Type II secretion system (T2SS), also known as the general secretory pathway, operates via a 2-step mechanism in which proteins are first translocated into the periplasm via the Sec or Tat pathways and then secreted through an outer membrane channel (Cianciotto, 2005, Cianciotto and White, 2017, Galan et al., 2014, Sandkvist, 2001a, Sandkvist, 2001b). The Type V secretion system (T5SS), also known as the autotransporter system, represents a simple mechanism in which the effector protein itself carries the machinery for secretion (da Mata Madeira et al., 2016, Gawarzewski et al., 2013, Meuskens et al., 2019). The Type IX secretion system (T9SS), primarily found in Bacteroidetes, is specialized for the secretion of proteins involved in virulence and pathogenesis delivering enzymes and immune-modulating factors (Gorasia et al., 2020, Nakayama, 2015, Polishchuk et al., 2025). More recently, the Type XI secretion system (T11SS) has been identified as a novel class of bacterial outer membrane proteins that transport cargo from the periplasmic space to the extracellular environment (Grossman et al., 2021, Grossman et al., 2025).

From an epigenetic perspective, injection systems that mediate cytosolic delivery, notably the T3SS, T4SS, T6SS, and T7SS, are most relevant, as they allow bacterial effectors to access intracellular compartments associated with chromatin regulation.

## THE HOST EPIGENOME

The host epigenome has recently emerged as a critical and dynamic interface in host-pathogen interactions (Arbibe, 2008). The epigenome provides an additional regulatory layer beyond the genetic code, primarily through chromatin modifications that control the accessibility of the transcriptional machinery to DNA (Inbar-Feigenberg et al., 2013, Mazzio and Soliman, 2012, Hamilton, 2011). Central to this regulation are post-translational histone modifications (PTMs)—including acetylation, methylation, phosphorylation, ubiquitination, and lactylation—that influence nucleosome structure and chromatin compaction (Cavalieri, 2021, Chen et al., 2024, Millan-Zambrano et al., 2022; Sadakierska-Chudy and Filip, 2015). These PTMs are dynamically written, erased, and read by specific epigenetic regulators, enabling the host to maintain gene expression programs, mount rapid responses to environmental cues, and establish long-term transcriptional memory (Trevino et al., 2015).

### Bacterial Infection and the Host Epigenome

The interaction between invading bacteria and the host immune system extends beyond classical signaling cascades, encompassing epigenetic regulation as an additional layer of control (Arbibe, 2008). Early immunological studies primarily focused on pathways such as NF-κB and MAPK that activate transcription factors and initiate gene expression (Caamano and Hunter, 2002, Gomez-Martin et al., 2010, Sha, 1998, Vallabhapurapu and Karin, 2009). However, recent findings reveal that the scope of host transcriptional regulation has expanded to include PTMs and chromatin remodeling during infection (Bierne et al., 2012; Lin et al., 2022, Zhang and Cao, 2019).

Pathogenic bacteria exploit the host epigenetic landscape through 2 principal strategies (Fig. 1). First, they directly modify host chromatin by delivering effector proteins that possess intrinsic histone-modifying activities. Second, they indirectly modulate histone modifications by deploying effectors, toxins, or metabolites that influence host signaling pathways governing chromatin-modifying enzymes. These direct and indirect mechanisms collectively enable pathogens to repress antimicrobial gene expression or, conversely, to activate inflammatory programs that facilitate infection, colonization, and dissemination (Zhang and Cao, 2019).

Importantly, such bacterial-induced epigenetic changes are not always transient. In some cases, chromatin remodeling leaves a long-lasting imprint on the host chromatin and transcriptional programs. These alterations can modify responses to subsequent infections or promote chronic inflammatory and immunosuppressive states (Bierne et al., 2012; Pereira et al., 2016). Consequently, epigenetic reprogramming should not be regarded as a mere by-product of infection but rather as a central strategy through which bacteria manipulate host immunity and shape disease outcomes.

### Bacterial Regulation of Host Histone Acetylation

#### Histone Acetylation

**Fig. 3:**
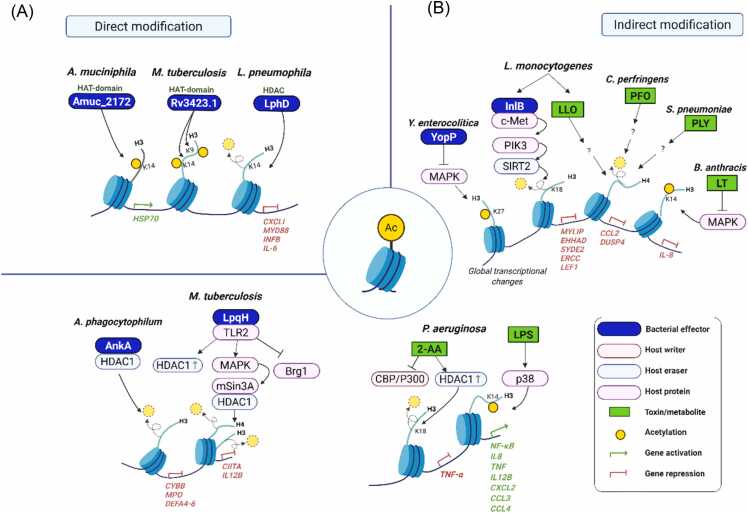
Bacterial regulation of host histone acetylation. (A) Direct modification: several bacterial effectors exhibit intrinsic enzymatic activities—such as histone acetyltransferase (HAT) or histone deacetylase (HDAC)—that directly add or remove acetylation marks on host histones. (B) Indirect histone modification via utilization of host acetylation machinery: some pathogens regulate host histone acetylation by recruiting chromatin-modifying enzymes or inducing their expression. Additionally, other pathogens indirectly modulate host signaling pathways, thereby altering histone acetylation dynamics and reprogramming host gene expression.

Histone acetylation is a key chromatin modification that regulates transcriptional accessibility and gene activation (Charidemou and Kirmizis, 2024; Grabiec and Potempa, 2018; Rahman et al., 2004). By neutralizing the positive charge of lysine residues, acetylation disrupts histone-DNA interactions and facilitates recruitment of the transcriptional machinery (Narita et al., 2019, Shvedunova and Akhtar, 2022). Pathogenic bacteria have evolved diverse and sophisticated mechanisms to manipulate host histone acetylation, exploiting it as a regulatory node to modulate immune responses in ways that enhance bacterial survival and persistence (Fig. 3 and Table 1).

**Table 1:** Bacterial regulation of host histone acetylation

Effector (E)/toxin (T)/ metabolite (M)	Bacterium	Secretion system/ secretion mechanism	Effector function	Host proteins involved	Histone PTM outcome	Reference
*Direct histone modification*						
Amuc_2172 (E)	*Akkermansia muciniphila*	Extracellular vesicles	HAT		↑ H3K14ac	Jiang et al. (2023),
Rv3423.1 (E)	*Mycobacterium tuberculosis*	T7SS	HAT		↑ H3K9/K14ac	Jose et al. (2016)
LphD (E)	*Legionella pneumophila*	T4SS	HDAC		↓ H3K14ac	Schator et al. (2023)

*Indirect histone modification*						
AnkA (E)	*Anaplasma phagocytophilum*	T4SS		HDAC1	↓ H3ac	Garcia-Garcia et al., 2009, Rennoll-Bankert et al., 2015
LpqH (E)	*M tuberculosis*			TLR2, Brg1	↓ H3ac, ↓ H4ac	Hamon and Cossart, 2008, Pennini et al., 2006; Wazahat et al. (2024)
InlB (E)	*Listeria monocytogenes*	Surface protein		c-Met, PIK3, and SIRT2	↓ H3K18ac	Eskandarian et al., 2013, Schator et al., 2021
LLO (T)	*L monocytogenes*	Sec-dependent pathway (type II-like)			↓ H4ac	Hamon et al. (2007)
PLY (T)	*Streptococcus pneumoniae*	Autolysis/cell wall turnover			↓ H4K9ac	Hamon et al. (2007)
PFO (T)	*Clostridium perfringens*	Sec-dependent pathway			↓ H4ac	Hamon et al. (2007)
LT (T)	*Bacillus anthracis*	T2SS		MAPK	↓ H3K14ac	Bardwell et al., 2004, Dong and Hamon, 2020
2-AA (M)	*Pseudomonas aeruginosa*	Quorum-sensing metabolite		CBP/p300, HDAC1	↓ H3K18ac	Bandyopadhaya et al. (2016)
LPS (T)	Gram-negative bacteria	Integral outer membrane component		p38	↑ H3K14ac	Rigillo et al., 2018, Saccani et al., 2002
YopP	*Yersinia enterocolitica*	T3SS		MAPK	Global changes in H3K27ac	Bekere et al. (2021)

2-AA, 2-aminoacetophenone; HAT, histone acetyltransferase; HDAC, histone deacetylase; LLO, listeriolysin; PFO, perfringolysin; PLY, pneumolysin.

#### Bacterial Effectors Directly Modulating Histone Acetylation

Several bacterial pathogens have evolved effectors that directly manipulate host histone acetylation to reprogram gene expression. *Akkermansia muciniphila* secretes Amuc_2172, a putative histone acetyltransferase effector that elevates H3K14ac and induces stress-response genes such as *HSP70*, which in turn enhances CD8+ T-cell activation and mucosal immune surveillance. Amuc_2172 shows structural similarity to members of the GCN5-related N-acetyltransferase superfamily, including a conserved central β-sheet and Motif A, which is required for acetyl-CoA binding and enzyme activity (Jiang et al., 2023, Mei et al., 2025). The *M tuberculosis* protein Rv3423.1 acts as a histone acetyltransferase mimic that is secreted via the T7SS and translocates to the macrophage nucleus, where it associates with chromatin and promotes H3K9 and/or H3K14 acetylation. Rv3423.1 is an unusually small protein predicted to adopt an acetyltransferase-like fold with a putative acetyl-CoA-binding site, despite the absence of a classical signal peptide or a recognizable nuclear localization sequence (Jose et al., 2016).

In contrast, *L pneumophila*, the causative agent of Legionnaires’ disease, relies on its T4SS to deliver numerous effectors that reprogram host transcription (Lockwood et al., 2022). Among these effectors, LphD functions as a eukaryotic-like histone deacetylase (HDAC) that removes acetyl groups from H3K14ac, thereby repressing the expression of innate immune response genes such as *CXCL1*, *MyD88*, *IFNB1*, and *IL-6* in infected human macrophages (Schator et al., 2023). Consistent with this activity, LphD is a 424-amino-acid protein predicted to adopt an HDAC-like fold. AlphaFold-based structural modeling indicates conservation of the Zn²⁺-dependent catalytic core, despite limited overall sequence similarity to host HDACs (Schator et al., 2023). LphD acts in concert with another *Legionella* effector, RomA (Rolando et al., 2013), which deposits the repressive H3K14me3 mark. This coordinated activity establishes an epigenetic mechanism in which an active acetyl mark is erased and replaced by a repressive methyl mark. Together, LphD and RomA target host chromatin via the KAT7 complex to fine-tune H3K14 acetylation and methylation, ultimately facilitating *L pneumophila* intracellular replication.

#### Indirect Histone Modification via Utilization of Host Histone Acetylation Machinery

Rather than encoding histone-modifying enzymes themselves, some pathogens indirectly modulate histone acetylation by deploying effectors, toxins, or metabolites that manipulate the host’s histone acetylation machinery, thereby reprogramming host gene expression to favor infection.

*Anaplasma phagocytophilum*, an intracellular bacterium, employs the effector AnkA via T4SS to reprogram host chromatin (Rennoll-Bankert and Dumler, 2012). Upon translocation into the host nucleus, AnkA binds to AT-rich DNA regions and recruits HDAC1 to the promoters of immune defense genes such as *CYBB*, *MP*O, and *DEFA4 to 6*, resulting in H3 deacetylation and transcriptional repression (Garcia-Garcia et al., 2009, Rennoll-Bankert et al., 2015).

Similarly, *M tuberculosis* manipulates host histone acetylation through its cell wall lipoprotein LpqH (Garcia-Garcia et al., 2009). LpqH interacts with TLR2 on macrophages and activates MAPK signaling, which in turn decreases histone H3 and H4 acetylation at the *CIITA* locus. This effect is accompanied by reduced recruitment of Brg1, a core component of the SWI/SNF chromatin remodeling complex (Pennini et al., 2006). Consistent with these findings, Chandran et al. reported that *M tuberculosis* infection increases HDAC1 expression (Chandran et al., 2015).

*Listeria monocytogenes* also subverts host acetylation by inducing H3K18 deacetylation. Its surface protein InlB activates PI3K signaling via the c-Met receptor, leading to the chromatin recruitment of the NAD⁺-dependent deacetylase SIRT2 (Eskandarian et al., 2013, Schator et al., 2021). Once recruited to chromatin, SIRT2 removes H3K18ac at the promoters of inflammatory genes such as *MYLIP*, *EHHADH*, *SYDE2*, *ERCC5*, and *LEF1*, leading to their transcriptional repression and the establishment of a host environment favorable for intracellular survival (Eskandarian et al., 2013).

Together, these findings highlight that bacterial effectors can exploit the host histone acetylation machinery to repress immune-related genes—primarily by recruiting or modulating host deacetylases and chromatin remodelers, thereby creating a more permissive intracellular niche for infection.

Beyond effector proteins, bacterial toxins and metabolites also modulate host histone acetylation indirectly by altering signaling pathways that control chromatin-modifying enzymes.

Cholesterol-dependent cytolysins—listeriolysin O (LLO), perfringolysin O (PFO), and pneumolysin (PLY)—produced by *L monocytogenes*, *Clostridium perfringens*, and *Streptococcus pneumoniae,* respectively, trigger pan-H4 deacetylation (Hamon et al., 2007). The resulting chromatin compaction represses the transcription of cytokine genes such as *CCL2*, *DUSP4*, *IRF3*, and *EGR1*, thereby dampening host immune responses and facilitating bacterial immune evasion.

*Bacillus anthracis* produces a bipartite exotoxin consisting of protective antigen (PA) and lethal factor, which together form the lethal toxin (LT) (Altmann, 2015, Bachran and Leppla, 2016, Little et al., 1996). Delivered into host cells via protective antigen-mediated endocytosis, LT acts as a metalloprotease that cleaves MAPK kinases, thereby inhibiting MAPK signaling (Bardwell et al., 2004, Dong and Hamon, 2020). This interference suppresses p38-mediated phosphorylation of H3S10 and the associated H3K14 acetylation at the IL-8 promoter in TNF-α-stimulated epithelial cells. While NF-κB activation itself is preserved, LT prevents NF-κB recruitment to the IL-8 locus by disrupting chromatin remodeling, ultimately repressing proinflammatory cytokine expression (Dong and Hamon, 2020, Raymond et al., 2009).

Lipopolysaccharide (LPS), a major component of Gram-negative bacterial cell walls, exerts broad effects on host histone modifications (Chiariotti et al., 2016). LPS induces H3K14ac and H3S10 phosphorylation at cytokine and chemokine promoters through p38 MAPK activation (Saccani et al., 2002). This enhances NF-κB recruitment and drives strong inflammatory transcription (ie, *IL-8*, *TNF*, *IL-12B*, *CXCL2*, *CCL3*, and *CCL4*) representing the canonical pathway by which innate immune recognition of bacterial components is linked to chromatin remodeling and effective immune activation. LPS-induced increase in H3S10phK14ac occurs mainly in neurons and reactive microglia, regulating neuronal and microglial neuroinflammatory (Rigillo et al., 2018).

Finally, the small volatile quorum-sensing (QS) molecule 2-aminoacetophenone (2-AA) serves as a key metabolite promoting host tolerance to bacterial burden. Produced abundantly by *Pseudomonas aeruginosa* and other pathogens in infected tissues, 2-AA synthesis is regulated by QS, a population density-dependent communication system controlling virulence. In acutely exposed host cells, 2-AA activates CBP/p300, which associates with NF-κB p65 and enhances its acetylation. In contrast, in 2-AA-tolerized cells, the p65-CBP/p300 interaction is disrupted, while p50-HDAC1 binding increases, leading to transcriptional repression of proinflammatory genes. These findings reveal how a bacterial QS molecule can link intracellular signaling to epigenetic reprogramming of inflammation-related genes, thereby facilitating host to tolerate long-term bacterial presence (Bandyopadhaya et al., 2016; Bandyopadhaya et al., 2017).

#### Genome-Wide Remodeling of Host Histone Acetylation During Bacterial Infection

Recent studies have extended analyses of bacterial effects on host histone modifications to the genome-wide level. A representative example is *Yersinia enterocolitica* infection, in which the T3SS induces extensive chromatin remodeling in primary human macrophages. Approximately 14,500 genomic loci show altered histone modification patterns following infection, with H3K27ac accounting for nearly 43% of affected regions (Bekere et al., 2021). This remodeling is driven largely by the T3SS effector YopP, which indirectly alters host histone acetylation through inhibition of MAPK signaling rather than direct histone-modifying activity. Genome-wide profiling reveals suppressed H3K27ac at inflammatory and type I interferon–associated genes, alongside relative preservation of acetylation at Rho GTPase-related genes, a pattern that correlates with reduced inflammatory gene expression and sustained transcription of cytoskeletal regulatory pathways.

In the context of *M tuberculosis* infection, a histone acetylome-wide association study analyzed H3K27 acetylation in peripheral blood granulocytes and monocytes from individuals with active tuberculosis and healthy controls (Del Rosario et al., 2022). More than 2,000 differentially acetylated loci were identified in each cell type, with the findings reproduced across independent discovery, validation, and longitudinal cohorts. Infection-associated changes in H3K27 acetylation correlated with differential gene expression and were enriched near immune-related genes, including potassium channel genes such as *KCNJ15*. Functional analyses showed that KCNJ15 expression promotes potassium influx, enhances apoptosis of infected cells, and facilitates intracellular clearance of *M tuberculosis* in vitro, linking histone acetylation changes to host defense-relevant cellular outcomes.

In *L pneumophila*-infected human macrophages, chromatin profiling revealed increased histone H4 acetylation at a defined subset of promoters (Du Bois et al., 2016). One well-characterized example is the *TNFAIP2* locus, where enhanced H4 acetylation is detected following infection. This change correlates with increased *TNFAIP2* transcription in infected macrophages and epithelial cells in an NF-κB-dependent manner. Functional analyses indicate that TNFAIP2 expression contributes to intracellular replication of *L pneumophila*, illustrating how infection-associated histone acetylation at specific loci can shape host transcriptional outputs and host-pathogen interactions.

### Bacterial Regulation of Host Histone Methylation

#### Histone Methylation

**Fig. 4:**
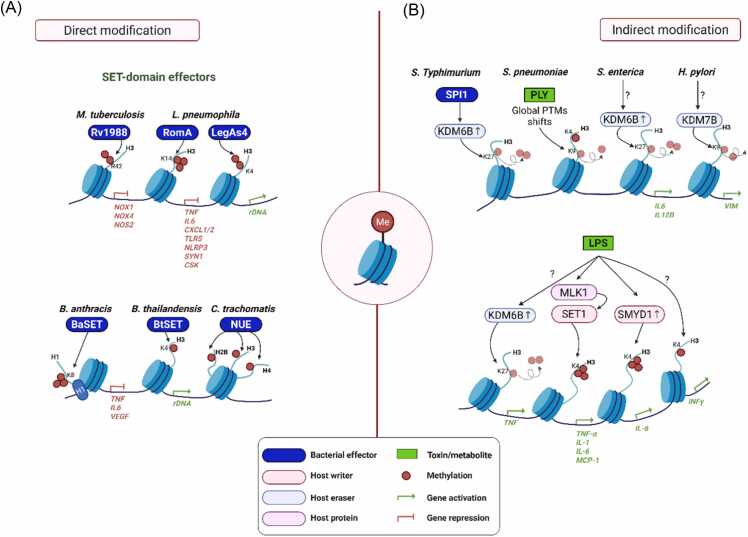
Bacterial regulation of host histone methylation. (A) Direct modification: various bacterial effectors harbor SET domains with histone methyltransferase activity and directly add methylation marks to host histones, resulting in activation or repression depending on the targeted amino acid residue. (B) Indirect histone modification via utilization of host methylation machinery: some pathogens regulate host histone methylation indirectly by modulating signaling pathways, thereby inducing the expression or activation of host histone methyltransferases and demethylases.

Histone methylation is a major epigenetic mechanism regulating transcriptional states, with its effects determined by the modified lysine or arginine residue and degree of methylation (mono-, di-, or tri-) (Lan and Shi, 2009, Sadakierska-Chudy and Filip, 2015, Zhang et al., 2012b). Unlike the more transient nature of acetylation, methylation marks are stable and serve as long-term indicators of gene activation or repression (Lubin et al., 2011, Razin and Cedar, 1991). Pathogenic bacteria exploit this stability in a manner similar to their manipulation of histone acetylation, using methylation as a regulatory mechanism to reconfigure host chromatin, modulate immune gene expression, and promote bacterial survival and persistence (Arbibe, 2008, Gomez-Diaz et al., 2012) (Fig. 4 and Table 2).

**Table 2:** Bacterial regulation of host histone methylation

Effector (E)/toxin (T)/ metabolite (M)	Bacterium	Secretion system/ secretion mechanism	Effector function	Host proteins involved	Histone PTM outcome	References
*Direct histone modification*						
Rv1988 (E)	*M tuberculosis*	Tat (Twin-arginine translocation) system	SET-domain methyltransferase		↑ H3R42me2	Yaseen et al. (2015)
RomA (E)	*L pneumophila*	T4SS	SET-domain methyltransferase		↑ H3K14me3	Rolando et al. (2013)
LegAS4 (E)	*L pneumophila*	T4SS	SET-domain methyltransferase		↑ H3K4me2	Bruggemann et al. (2006), Li et al. (2013)
BaSET (E)	*B anthracis*	Sec-dependent pathway	SET-domain methyltransferase		↑ H1K8me3	Mujtaba et al. (2013)
BtSET (E)	*Burkholderia thailandensis*	T6SS	SET-domain methyltransferase		↑ H3K4me	Li et al. (2013)
NUE (E)	*Chlamydia trachomatis*	T3SS	SET-domain methyltransferase		↑ H2Bme, ↑ H3me, ↑ H4me	Pennini et al. (2010)

*Indirect histone modification*						
SPI1 (E)	*Salmonella enterica*	General infection		KDM6B	↓ H3K27me3	Rana et al. (2021)
	*Helicobacter pylori*	General infection		KDM7B	↓ H3K9me2	Li et al. (2017)
PLY (T)	*S pneumoniae*	Autolysis/cell wall turnover		?	↓ H3K9me2, ↑ H3K4me1, ↓ H3K27me2	Chevalier et al., 2024, Cole et al., 2021
LPS (T)	Gram-negative bacteria	Integral outer membrane component		Elevates SAM production	↑ H3K6me3	Yu et al. (2019)
				KDM6B	↓ H3K27me3	He et al., 2020, Liu et al., 2018
				MLK1, SET1	↑ H3K4me3	Yu et al. (2017)
				SMYD1	↑ H3K4me3	Shamloul et al. (2021)
				?	↑ H3K4me1	Rasid et al. (2019)

#### Bacterial Effectors Directly Modulate Histone Methylation

Several bacterial pathogens have evolved effector proteins with intrinsic histone methyltransferase activity, enabling them to directly manipulate host chromatin and transcriptional programs. Among the effectors characterized to date, many contain SET domains, a conserved catalytic fold associated with lysine methylation and shared with eukaryotic histone lysine methyltransferases (Herz et al., 2013). *L pneumophila* employs multiple SET-domain effectors to remodel host chromatin. RomA, delivered through the Type IV secretion system, catalyzes trimethylation of H3K14 (H3K14me3), a previously unreported histone modification in mammals (Rolando et al., 2013). This modification contributes to transcriptional repression during infection. Another *Legionella* effector, LegAS4, specifically dimethylates H3K4 at rDNA promoters (Bruggemann et al., 2006). By interacting with heterochromatin proteins HP1α and HP1γ, LegAS4 reactivates rDNA transcription and enhances ribosome biogenesis, thereby supporting bacterial replication within host cells (Li et al., 2013).

SET-domain effectors targeting host chromatin have also been described in other bacterial species. *B anthracis* secretes BaSET, which trimethylates histone H1 and represses NF-κB-driven genes such as *TNFα*, *IL-6*, and *VEGF* (Mujtaba et al., 2013). The loss of BaSET abolishes virulence in murine models, highlighting its essential role in immune evasion. Similarly, *Burkholderia thailandensis* translocates BtSET, which promotes H3K4 methylation at rDNA loci, leading to increased rRNA synthesis and intracellular proliferation (Li et al., 2013). *Chlamydia trachomatis* produces NUE, a broad-spectrum SET-domain methyltransferase secreted via the T3SS. NUE associates with host chromatin and methylates multiple histones, including H2B, H3, and H4, while also undergoing automethylation to enhance its enzymatic activity (Pennini et al., 2010). This versatility suggests that NUE provides *Chlamydia* with a means of long-term epigenetic reprogramming during persistent infection.

In addition to lysine methylation mediated by SET-domain proteins, a limited number of bacterial effectors have been shown to catalyze arginine methylation through mechanistically distinct pathways. A representative example is *M tuberculosis* Rv1988 (Erm37), a Tat-secreted effector that dimethylates H3R42 (H3R42me2) (Yaseen et al., 2015). This repressive modification silences key antimicrobial genes, including *NOX1*, *NOX4*, and *NOS2* in THP-1 macrophage cells, thereby suppressing host oxidative and nitrosative defenses.

Together, these findings illustrate a recurring theme: pathogenic bacteria exploit histone methyltransferase effectors to reshape host transcriptional landscapes. By introducing novel or aberrant methylation marks, these effectors silence antimicrobial responses, reactivate ribosome biogenesis, and promote intracellular survival, underscoring histone methylation as a central battleground in host-pathogen interactions.

#### Indirect Histone Modification via Utilization of Host Histone Methylation Machinery

*Salmonella enterica* serovar Typhimurium manipulates host histone methylation by inducing the histone demethylase KDM6B, leading to a global reduction in the repressive mark H3K27me3. Upon infection, *Salmonella* Typhimurium transcriptionally and translationally upregulates KDM6B, which is specifically recruited to promoters of WNT pathway genes, including *WNT3* and *PPARδ*, where it mediates H3K27me3 demethylation through SPI1 effectors (Rana et al., 2021). Similarly, *H pylori* modulates the host methylation machinery by upregulating the histone demethylase KDM7B (also known as PHD finger protein 8, PHF8), which targets H3K9me2 for demethylation. PHF8 is recruited to the vimentin promoter via its physical interaction with β-catenin, enhancing vimentin transcription in gastric cancer cells. This PHF8-β-catenin-vimentin axis represents a novel epigenetic mechanism by which *H pylori* contributes to gastric cancer progression (Li et al., 2017).

Bacterial toxins such as PLY and LPS actively exploit host signaling pathways to orchestrate immune gene expression. In human monocyte-derived macrophages, Cole et al. (2021) demonstrated, using either the wild-type or a PLY-deficient (Δ*ply*) strain, that PLY drives global shifts in histone post-translational modifications, specifically increasing H3K4me1 and H4K16ac, while reducing H3K9me2 and H3K79me2, in a PLY-dependent fashion upon 3 hours of bacterial challenge. These alterations correspond to attenuated early induction of proinflammatory cytokines such as TNF-α and IL-6, suggesting that Ply-mediated chromatin remodeling suppresses transcriptional activation of key immune genes (Cole et al., 2021). In contrast, in epithelial cells, Chevalier et al*.* reported that *S pneumoniae* infection induces a persistent increase in H3K4me2 across enhancer regions, which remains elevated even after bacterial clearance and is inherited through cell division. This dimethylation mark is unique compared with H3K4me1 or H3K4me3 and is associated with altered transcriptional responsiveness upon secondary infection, including enhanced bacterial adherence and metabolic remodeling (Chevalier et al., 2024). Taken together, these studies support a model in which *S pneumoniae* infection contributes to immune evasion by skewing the host histone PTM landscape in macrophages by suppressing proinflammatory gene activation through removal of repressive and addition of permissive marks, and in epithelial cells, by imprinting a stable H3K4me2 “memory” at enhancers that biases gene expression toward a more permissive state during subsequent exposures.

Analogously, LPS orchestrates extensive chromatin remodeling to shape inflammatory gene expression. In macrophages, LPS recruits MKL1 to NF-κB target promoters, where it collaborates with SET1 to deposit activating H3K4me3 marks, reinforcing p65-dependent proinflammatory gene transcription such as *TNF-α*, *IL-1*, *IL-6*, and *MCP*-*1*. In endothelial cells, LPS elevates the lysine methyltransferase SMYD1, which promotes IL-6 transcription through NF-κB activation and H3K4me3 enrichment at the *IL-6* promoter (Shamloul et al., 2021). Systemically, LPS exposure reprograms innate lymphocytes, with NK cells acquiring long-lasting memory-like responses dependent on an H3K4me1-marked enhancer at the *Ifng* locus (Rasid et al., 2019). LPS also induces the histone demethylase KDM6B, which removes repressive H3K27me3 from inflammatory gene promoters (He et al., 2020, Liu et al., 2018), and promotes S-adenosylmethionine production, enabling H3K36me3 deposition at the *IL-1B* promoter (Yu et al., 2019). Together, these mechanisms demonstrate that LPS remodels host histone methylation landscapes by enriching activating marks, removing repressive marks, and integrating with NF-κB signaling to drive potent and sustained inflammatory programs.

In summary, bacterial effectors and toxins profoundly influence host histone methylation either by enhancing activating marks to amplify inflammation or by modulating repressive marks to evade immune detection. Through these multifaceted strategies, pathogens effectively rewire the host’s epigenetic circuitry to promote persistence, immune tolerance, or recurrent infection.

### Bacterial Regulation of Other Histone Modifications

**Fig. 5:**
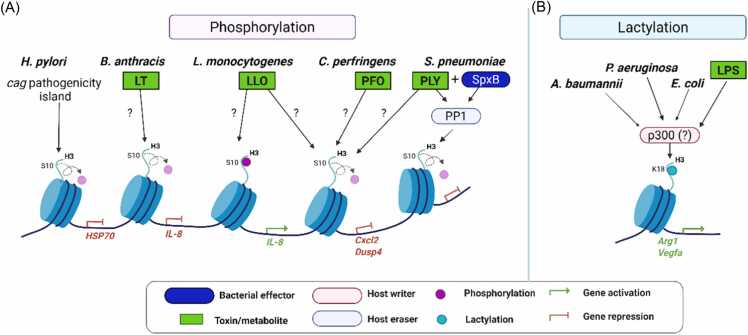
Bacterial regulation of host histone phosphorylation and lactylation. Bacterial infection is associated with changes in histone phosphorylation and lactylation, largely through indirect mechanisms. (A) Histone phosphorylation, in which bacterial toxins or effectors modulate host signaling pathways or phosphatase activity, leading to context-dependent changes in histone H3S10 phosphorylation. (B) Histone lactylation, in which infection-induced metabolic changes, such as increased intracellular lactate, are associated with altered histone lactylation, potentially through effects on host acetyltransferases such as p300.

In addition to acetylation and methylation, bacteria can modulate other PTMs, such as phosphorylation and lactylation, to influence host chromatin states and immune responses (Banerjee and Chakravarti, 2011, Hu et al., 2024, Rossetto et al., 2012, Xie et al., 2022, Zhang et al., 2019). These modifications serve as dynamic switches for chromatin accessibility and transcriptional control, representing key interfaces in host-pathogen interactions (Fig. 5 and Table 3).

**Table 3:** Bacterial regulation of host other histone modifications

Effector (E)/ toxin (T)/ metabolite (M)	Bacterium	Secretion system/ secretion mechanism	Effector function	Host proteins involved	Histone PTM outcome	References
*Phosphorylation*						
LLO (T)	*L monocytogenes*	General infection (bacterial entry into the cytoplasm of endothelial cells)	?	NOD1	↑ H3S10p	Opitz et al., 2006, Schmeck et al., 2005
LLO (T)	*L monocytogenes*	Sec-dependent pathway (type II-like)			↓ H3S10p	Hamon et al. (2007)
PFO (T)	*C perfringens*	Sec-dependent pathway			↓ H3S10p	Hamon et al. (2007)
LT (T)	*B anthracis*				↓ H3S10p	Raymond et al. (2009)
*cag pathogenicity island*	*H pylori*				↓ H3S10p	Ding et al. (2010)
PLY (T), SpxB (E)	*S pneumoniae*	Autolysis/cell wall turnover		PP1	↓ H3S10p	Dong et al., 2020, Hamon et al., 2007

*Lactylation*						
LPS (T)	*P aeruginosa, Escherichia coli, Acinetobacter baumannii*				↑ pan-Kla↑ H3K18la	Zhang et al. (2019)

LLO, listeriolysin; PFO, perfringolysin; PLY, pneumolysin.

#### Histone Phosphorylation

Histone phosphorylation is commonly linked to rapid cellular responses, including DNA damage signaling and mitogen-induced transcriptional activation (Healy et al., 2012, Sawicka and Seiser, 2014, Sharma and Hendzel, 2019). Several bacterial pathogens have been shown to modulate this modification during infection, particularly at histone H3 serine 10 (H3S10), with consequences for host inflammatory gene expression.

During infection with *L monocytogenes*, phosphorylation of H3S10 is induced at promoters of proinflammatory genes such as *IL-8*, coinciding with increased IL-8 production following bacterial entry into the cytoplasm of endothelial cells (Opitz et al., 2006, Schmeck et al., 2005). Subsequent work demonstrated that wild-type *L monocytogenes*, but not a LLO-deficient mutant, stimulates IL-8 expression through NOD1-dependent activation of MAPK signaling in human endothelial cells (Opitz et al., 2006, Schmeck et al., 2005). In contrast, during early stages of infection with extracellular bacteria, *L monocytogenes* induces pronounced dephosphorylation of histone H3 and deacetylation of histone H4 in an LLO-dependent manner (Hamon et al., 2007). H3S10 dephosphorylation was also observed following exposure to other cholesterol-dependent cytolysins, including PLY and PFO. These decreases in histone phosphorylation and acetylation correlate with reduced transcription of a subset of immune genes, such as *CXCL2* and *DUSP4* (Hamon et al., 2007).

A similar suppression of histone phosphorylation has been reported for *B anthracis*. Active LT inhibits cytokine production and neutrophil recruitment in the lung, both in vivo and in pulmonary epithelial cells. This effect is associated with blockade of H3S10 phosphorylation and reduced accessibility of NF-κB p65 to promoters of inflammatory genes, including *IL-8*, leading to diminished transcriptional activation (Raymond et al., 2009).

Wild-type *H pylori* infection induces time- and dose-dependent dephosphorylation of histone H3S10 and decreases acetylation of H3K23 in gastric epithelial cells. These effects are independent of CagA, VacA, or flagellar function, but require an intact *cag* pathogenicity island (cagPAI). The resulting histone modifications are linked to altered expression of genes such as *c-JUN* and *HSP70*, suggesting that *H pylori* modulates host chromatin states to promote persistent infection and pathogenesis (Ding et al., 2010, Schator et al., 2021).

More recently, infection of respiratory epithelial cells with *S pneumoniae* was shown to elicit a characteristic epigenetic response marked by strong dephosphorylation of H3S10. This process depends on the combined activity of PLY- and SpxB-derived hydrogen peroxide, which activates host protein phosphatase 1 and promotes H3S10 dephosphorylation (Dong et al., 2020).

Taken together, these studies indicate that multiple bacterial pathogens modulate host H3S10 phosphorylation through toxin- and signaling-dependent mechanisms. Depending on the infection context, such modulation is associated with either enhanced or reduced transcription of inflammatory genes. In the case of *L monocytogenes*, opposing effects on H3S10 phosphorylation have been observed during intracellular invasion vs extracellular exposure, highlighting the context-dependent nature of LLO-mediated regulation and the need for further work to define the determinants governing these divergent outcomes.

#### Histone Lactylation

Histone lactylation is a recently described post-translational modification that links cellular metabolic state to chromatin regulation (Hu et al., 2024). This modification involves the covalent attachment of a lactyl group to lysine residues and is thought to occur under conditions of elevated intracellular lactate. Current evidence indicates that the acetyltransferase p300 can catalyze histone lactylation in vitro and in cells, particularly when lactate levels are increased as a result of enhanced glycolytic activity (Zhang et al., 2025, Zong et al., 2025).

Initial studies demonstrated that bacterial challenge stimulates glycolysis-dependent lactate production in macrophages, which is accompanied by increased histone lactylation and altered gene expression profiles (Zhang et al., 2019). Stimulation with *Acinetobacter baumannii*, *P aeruginosa*, *Escherichia coli*, or LPS induces classical macrophage activation together with a metabolic shift toward aerobic glycolysis. During the later phase of this response, accumulated intracellular lactate serves as a substrate for histone lysine lactylation. Increased histone lactylation is enriched at promoter regions of genes associated with tissue repair and homeostasis, including *Arg1*, and correlates with transcriptional activation of these loci (Zhang et al., 2019). These findings suggest that histone lactylation may contribute to the temporal regulation of macrophage gene expression during infection, coinciding with a transition from an early proinflammatory state toward a more reparative transcriptional program.

Despite these observations, the extent to which bacterial pathogens modulate histone lactylation in vivo, as well as the specificity and functional consequences of this modification across different infection contexts, remains incompletely defined. Further studies will be required to clarify the regulatory mechanisms and biological significance of histone lactylation during host-pathogen interactions.

## DISCUSSION

Accumulating evidence indicates that bacterial pathogens influence host transcriptional programs not only through interference with signaling pathways but also through modulation of the host epigenome. The studies discussed here can be organized along a common conceptual axis: direct chromatin modification by bacterial effectors vs indirect modulation of host epigenetic states through altered signaling and metabolism. This framework helps reconcile diverse observations across pathogens and infection contexts.

In the direct mode, several pathogens deliver effectors with intrinsic histone-modifying activity, including acetyltransferases and methyltransferases, that target specific histone residues and genomic regions. These activities are often highly selective, such as preferential modification of H3K14 or rDNA-associated histones. Such specificity suggests that bacterial effectors exploit histone marks with strong regulatory leverage over immune responses or cellular biosynthetic capacity. Interestingly, multiple bacterial effectors with HAT-like activity converge on H3K14 acetylation. In host cells, H3K14ac is primarily deposited by the HBO1 (KAT7) and GCN5/PCAF complexes, which are involved in transcriptional activation, DNA replication, and stress responses (Johnsson et al., 2009, Lan and Wang, 2020). Whether bacterial HATs independently evolved to target this residue or share deeper structural or mechanistic similarities with host HAT complexes remains unclear. Addressing this question will require comparative structural and biochemical analyses to determine how bacterial enzymes achieve substrate specificity and whether common principles underlie chromatin susceptibility during infection.

Indirect epigenetic modulation arises when pathogens alter host signaling pathways or metabolic states that secondarily affect chromatin-modifying enzymes. Inhibition of MAPK or NF-κB signaling, toxin-mediated activation of host phosphatases, and infection-induced metabolic rewiring have each been associated with changes in histone acetylation, phosphorylation, or lactylation. In many cases, these indirect mechanisms account for widespread chromatin remodeling in the absence of direct bacterial histone-modifying activity, underscoring the central role of host regulatory networks as intermediaries between infection and epigenetic change.

Despite substantial progress, several important gaps remain. First, the temporal dynamics and reversibility of infection-induced epigenetic changes are poorly characterized. While some histone modifications appear transient and tightly linked to acute signaling events, others persist and may influence subsequent immune responses. Clarifying these dynamics will require longitudinal and single-cell approaches capable of resolving heterogeneous chromatin states over time. Second, the integration of histone modifications with signaling and metabolic pathways remains insufficiently understood, particularly with respect to how combinatorial epigenetic states are established and interpreted during infection. Third, most current studies focus on a limited number of model pathogens and host cell types, limiting the generalizability of proposed mechanisms across diverse microbial species and tissue environments. In addition, whether infection-induced epigenetic changes predominantly occur at specific loci or extend to broader genomic domains remains inadequately explored.

Future studies would benefit from experimental designs that combine genome-wide chromatin profiling with defined infection models, rather than relying on single-layer analyses. For example, integrating histone PTM profiling with chromatin accessibility measurements in the same infected cell populations would allow direct assessment of how bacterial effectors reshape regulatory elements over time. In parallel, coupling these datasets with transcriptomic and targeted metabolic measurements could help distinguish epigenetic changes driven by direct effector activity from those arising indirectly through infection-induced metabolic rewiring.

Another important direction will be the systematic dissection of effector-chromatin interactions, including determining how individual effectors influence the recruitment or activity of host chromatin-modifying enzymes at specific genomic loci. Extending such analyses beyond a limited set of well-studied histone marks and pathogens will be necessary to assess the generality of current models. Ultimately, these approaches may clarify how infection-associated epigenetic states are established and maintained, and whether they can be selectively reversed to restore host transcriptional and immune functions without directly targeting bacterial viability.

In summary, bacterial modulation of the host epigenome is best viewed as a continuum ranging from direct enzymatic modification of chromatin to indirect reprogramming mediated by altered host signaling and metabolism. Dissecting how these modes intersect in different infection contexts will be essential for understanding host-pathogen interactions and for identifying epigenetic vulnerabilities that may be exploited for therapeutic intervention.

## Author Contributions

**Kyunghwan Kim:** Writing – review & editing, Supervision, Funding acquisition, Conceptualization. **Shira Zelikman:** Writing – review & editing, Writing – original draft, Visualization, Conceptualization. **Sun-Ju Yi:** Writing – review & editing, Visualization, Conceptualization.

## Declaration of Competing Interests

The authors declare that they have no known competing financial interests or personal relationships that could have appeared to influence the work reported in this paper.
